# Adipositas und Diabetes mellitus Typ 2 (Update 2026)

**DOI:** 10.1007/s00508-026-02706-y

**Published:** 2026-04-30

**Authors:** Johanna M. Brix, Martin Clodi, Jürgen Harreiter, Friedrich Hoppichler, Florian W. Kiefer, Bianca-Karla Itariu, Verena Parzer, Michael Resl, Karin Schindler, Hermann Toplak, Thomas C. Wascher, Bernhard Ludvik

**Affiliations:** 1 1. Medizinische Abteilung mit Diabetologie, Endokrinologie und Nephrologie, Klinik Landstraße, Wien, Österreich; 2 Karl Landsteiner Institut für Adipositas und Stoffwechselerkrankungen, Wien, Österreich; 3 Abteilung für Innere Medizin, Konventhospital der Barmherzigen Brüder Linz, Linz, Österreich; 4 ICMR – Institute for Cardiovascular and Metabolic Research, Johannes Kepler Universität Linz, Linz, Österreich; 5 Abteilung für Innere Medizin mit Palliative Care, Landesklinikum Scheibbs, Scheibbs, Österreich; 6 Abteilung für Endokrinologie und Stoffwechsel, Universitätsklinik für Innere Medizin III, Medizinische Universität Wien, Wien, Österreich; 7 Abteilung für Innere Medizin, Krankenhaus der Barmherzigen Brüder Salzburg, Salzburg, Österreich; 8 SIPCAN – Initiative für ein gesundes Leben, Salzburg, Österreich; 9 Stoffwechselzentrum N12, Antonigasse 12, Wien, Österreich; 10 Nussdorfer Strasse 6/9, Wien, Österreich; 11 Universitätsklinik für Innere Medizin, Abteilung für Endokrinologie und Diabetologie, Medizinische Universität Graz, Graz, Österreich; 12 1. Medizinische Abteilung, Mein Hanusch-Krankenhaus, Wien, Österreich

**Keywords:** Medikamentöse Adiposithastherapie, Metabolische Chirurgie, Körperzusammensetzung, Ernährung, Pharmacological obesity therapy, Metabolic surgery, Body composition, Nutrition

## Abstract

Adipositas ist eine chronische Erkrankung mit einer Vielzahl an metabolischen, mechanischen und psychosozialen Komplikationen und einem hohen Risiko, an Prädiabetes bzw. in weiterer Folge an Diabetes mellitus Typ 2 zu erkranken. Sowohl die Wahl der antidiabetischen Therapie als auch der Begleittherapien nimmt vermehrt auf das Vorliegen der Adipositas Rücksicht. Die modernen GLP-1-Analoga sowie der kombinierte GIP/GLP-1-Agonist Tirzepatid nehmen einen wichtigen Stellenwert in der gemeinsamen Behandlung von Adipositas und Diabetes mellitus Typ 2 ein. Die metabolische Chirurgie ist derzeit bei an Diabetes mellitus Typ 2 erkrankten Menschen mit einem BMI > 30 kg/m^2^ indiziert, kann zur Diabetesremission beitragen, muss jedoch in ein entsprechendes, lebenslanges Betreuungskonzept eingebunden sein. Die Lebensstilmodifikation ist die Grundlage jeder Adipositastherapie. Aufgrund der Vielzahl an neuen Medikamenten sowie der vielen neuen Studien, bei denen versucht wurde, die relevantesten zu zitieren, erfolgten große Änderungen in diesem Kapitel im Vergleich zu 2023. Auch die Indikationen für eine metabolische Therapie wurden angepasst.

## Abstract (en)

Obesity is a chronic disease with a variety of metabolic, mechanical and psychosocial complications, including a high risk of developing prediabetes and subsequently type 2 diabetes mellitus. The choice of antidiabetic treatment as well as concomitant treatment, increasingly takes this into account. Modern glucagon-like peptide 1 (GLP-1) analogues and the combined gastric inhibitory polypeptide (GIP)/GLP‑1 agonist tirzepatide play an important role in the combined treatment of obesity and type 2 diabetes mellitus. Metabolic surgery is currently indicated for people with type 2 diabetes mellitus and a body mass index (BMI) > 30 kg/m^2^ and can contribute, at least in part, to diabetes remission but it must be integrated into an appropriate lifelong care plan. Every obesity therapy is always based on lifestyle modifications. Due to the large number of new drugs and the many new studies in which an attempt was made to cite the most relevant ones, major changes were made to this chapter compared to 2023. The indications for metabolic treatment were also adjusted.

Die chronische Erkrankung Adipositas ist der wohl wichtigste Risikofaktor für die Entwicklung eines Typ-2-Diabetes. Es gibt überzeugende Evidenz, dass die Behandlung von Adipositas die Progression von Prädiabetes zu Typ-2-Diabetes verzögern kann und bei der Therapie von Typ-2-Diabetes von großem Nutzen ist [1–4]. Bei Menschen mit Typ-2-Diabetes und Übergewicht oder Adipositas verbessert bereits eine geringe Gewichtsabnahme den Blutzuckerspiegel und verringert den Bedarf an blutzuckersenkenden Medikamenten, eine größere Gewichtsabnahme kann dazu beitragen, den HbA_1c_ und den Nüchternblutzucker erheblich zu senken, und kann eine Remission des Diabetes fördern [5–7].

In der EU sind 15 % der Menschen zwischen 16 und 74 Jahren von Adipositas und 50 % von Übergewicht betroffen [8]. Ein erhöhter BMI bedeutet eine erhöhtes Mortalitätsrisiko bei Männern und Frauen, wobei dies allerdings auch für Untergewicht gilt („J shaped curve“) [9]. Weltweit ist der größte Anteil des Typ-2-Diabetes der Adipositas zuzuordnen, so auch in Europa [10]. Diabetes und Adipositas zusammen erhöhen das Mortalitätsrisiko auf das 7‑Fache [11]. Die European Association for the Study of Obesity (EASO) hat sie zum „Gateway of ill Health“, also zu einem zentralen krankheitsbestimmenden Faktor, erklärt [12]. Die WHO bezeichnet die Adipositas als größtes globales chronisches Gesundheitsproblem, das neuerdings die Bedeutung der Malnutrition bei Weitem übertrifft [13, 14]. Neben den an anderer Stelle behandelten Themen soll der Typ-2-Diabetes im Folgenden aus diesem Blickwinkel betrachtet werden.

## Anthropometrie

Adipositas ist bis heute von der WHO definiert als BMI ≥ 30 kg/m^2^ [15]. Allerdings ist erwiesen, dass jeder BMI mit einem unterschiedlichen Körperfettanteil verbunden sein kann. Der BMI stellt jedoch lediglich ein populationsbezogenes Screening-Instrument dar und erlaubt keine zuverlässige Aussage über den individuellen Körperfettanteil oder den Gesundheitszustand einer Person. Zwar weisen fast 100 % der Personen mit einem BMI > 30 kg/m^2^ einen hohen Fettanteil auf, aber auch immerhin noch etwa ein Drittel der Normalgewichtigen [16]. Dies ist auf den häufigen Verlust der Muskelmasse und der Muskelkraft mit zunehmendem Alter oder Mangel an Muskelmasse (Sarkopenie) zurückzuführen, die eine ungünstige Fett-Muskel-Relation auch bei Normal- oder Übergewicht bedeutet [17–19]. Daher ist eine weitere anthropometrische Messung erforderlich, um eine korrekte Diagnose zu stellen. Es sollte bei jeder Person mit erhöhten BMI auch der Taillenumfang gemessen werden [20]. Besteht eine abdominelle Fettansammlung, ist das Risiko für Atherosklerose und vorzeitige Mortalität selbst bei normalem BMI erhöht [21]. Als Grenzwerte für den Taillenumfang gelten für eine kaukasische Bevölkerung 88 cm für Frauen und 102 cm für Männer [22]. Da andere ethnische Populationen (z. B. Asiaten) bei gleichem BMI eine größere Fettmasse aufweisen, werden für diese Kollektive andere Grenzwerte herangezogen, für weitere Ethnizitäten gibt es nur teilweise Grenzwerte (Tab. 1; [23, 24]). Die IDF (International Diabetes Federation) gibt für den Taillenumfang unterschiedliche Grenzwerte für unterschiedliche Regionen an, allerdings nicht für den BMI [24]. Da unterschiedliche Körpergrößen auch einen Einfluss auf den Taillenumfang haben können, wird, um eine korrekte Einschätzung angeben zu können, die Bestimmung der „Waist to Height Ratio“ – „Taillenumfang-zu-Körpergröße-Ratio“ empfohlen. Hier gilt ein Wert ab ≥ 0,5 mit einem erhöhten Risiko für Diabetes mellitus, metabolisches Syndrom und kardiovaskuläre Erkrankungen assoziiert, ein Wert von ≥ 0,6 als pathologisch [25]. Wenn möglich sollte auch eine Bioimpedanzanalyse als indirekte Methode zur Erfassung der fettfreien Masse bzw. der Körperzellmasse ergänzend erfolgen. Auch diese Methode hat Limitationen, ermöglicht aber eine gute Orientierung v. a. im Verlauf des Gewichtsmanagements [20]. Eine Methode zur Erfassung des Körperfettes ist die „dual energy X‑ray absorptiometry“ (DEXA; [26]). Aufgrund des Aufwandes und der hohen Kosten kommt diese aber zumeist nur in Studien zum Einsatz. Dies gilt auch für BODPOD, CT und MR-Untersuchungen.

**Tab. 1:** Grenzwerte von anthropometrischen Messungen abhängig von der Ethnizität

Ethnizität	Kaukasische Frauen/Männer	Asiatische Frauen/Männer
BMI, kg/m^2^	30	27,5
Taillenumfang, cm*	88/102	80/90
Waist/Height Ratio*	> 0,5	> 0,5

* Gültig in Menschen mit BMI < 35 kg/m^2^

## Implikationen für das Lebensstilmanagement

Lebensstilinterventionen stellen einen unverzichtbaren Bestandteil jedes Gewichts- und Gesundheitsmanagements dar und umfassen Ernährung, körperliche Aktivität, Schlaf und verhaltenstherapeutische Maßnahmen. Allein angewendet sind sie jedoch bei den meisten Menschen mit Adipositas nicht ausreichend, um eine nachhaltige Gewichtsreduktion oder Gewichtsstabilisierung zu erreichen [27]. Beim Gewichtsmanagement bevorzugen weltweit in allen Kulturen Frauen Ernährungsmaßnahmen und Männer Bewegungsmaßnahmen [28, 29]. Um eine Zunahme der Muskelmasse und eine Verminderung der Körperfettmasse zu erzielen, braucht man in der Regel aber beides. Einen besonders wichtigen Faktor stellt neben der Muskelmasse die Funktionalität dar, weswegen im Monitoring geeignete Parameter empfohlen werden (z. B. „Handgrip-Test“) [30]. Insbesondere im Alter ist die körperliche Fitness von großer prognostischer Bedeutung [31, 32].

Eine nachhaltige Veränderung der Ernährung ist ein wesentlicher Bestandteil jeder Gewichtsintervention. Dafür ist ein strukturiertes Vorgehen unter Einbindung spezialisierter Ernährungsfachkräfte erforderlich. Von Vorteil dürfte eine mediterrane Ernährung sein, sie konnte bei Menschen mit Diabetes die Notwendigkeit der Verordnung oraler Antidiabetika bei Neudiagnose reduzieren [33]. In der Ernährungstherapie sind heute individuell maßgeschneiderte Kostformen, die auch persönliche Präferenzen, Abneigungen, den kulturellen und religiösen Hintergrund sowie die individuelle ökonomische Situation in Betracht ziehen, zu erstellen. Supplemente mit definiertem Inhalt können im Ersatz einzelner oder mehrerer (meist 2) Mahlzeiten hilfreich sein („low calorie diets“) [34]. Für kurze Zeiträume können bei entsprechender Eignung der Patient:innen auch stark hypokalorische ketogene Kostformen („very low calorie diets [VLCDs]“) eingesetzt werden, die dann meist von „low calorie diets“ mit 1‑ bis 2‑mal täglichem Mahlzeitenersatz (LCDs) über längere Zeit gefolgt werden [35].

In den letzten Jahren hat das Intervallfasten zunehmend an Bedeutung gewonnen, da man als Betroffener hier nicht Kalorienzählen muss und es auch an individuelle Vorlieben anpassen kann. Außerdem ist es auch für Menschen mit Insulintherapie gut umsetzbar [36]. Hierbei gilt es, unterschiedliche Arten zu unterscheiden [37]:

- „Alternate-day fasting“ – hier isst man einen Tag und einen Tag isst man nicht (1:0).
- Intervallfasten‚ „time restricted eating“ – hier wechseln sich Zeiten der Nahrungseinschränkung mit Zeiten von Ad-libitum-Nahrungsaufnahme ab. Die beliebtesten Formen sind 16:8 (16 h fasten und 8 h Nahrungsaufnahme).
- Intermittierendes Fasten – hier werden z. B. 2 Fastentage pro Woche eingelegt, an denen maximal 500 kcal konsumiert werden.

Studien zeigen, dass auch Menschen mit Typ-2-Diabetes unter Insulintherapien von Intervallfasten bezüglich ihrer glykämischen Einstellung sehr profitieren, in einer 12-wöchigen Interventionsstudie konnte der HbA_1c_ um 2,8 % gesenkt werden (Ausgangs-HbA_1c_: 8,3 ± 1,1 %, mittlerer BMI 34,1 ± 4,5 kg/m^2^) ohne eine Zunahme von Hypoglykämien [38].

## Benefit von Gewichtsverlust in der Diabetestherapie

Nachdem die Mehrheit der Menschen mit Typ-2-Diabetes auch an Übergewicht oder Adipositas leidet, stellt die Gewichtsreduktion eine wichtige therapeutische Maßnahme bzw. das Therapieziel dar [39]. Dies gilt insbesondere, da die zugrunde liegenden Stoffwechselstörungen, die letztendlich zu Hyperglykämie führen, in der Regel bereits Jahrzehnte vor der Diagnose eines Typ-2-Diabetes auftreten und durch Gewichtszunahme, zentrale Adipositas und Insulinresistenz bedingt sind.

In der „Look AHEAD“-Studie wurden Menschen mit Typ-2-Diabetes (*n* = 5145) randomisiert, um den Effekt einer intensiven Lebensstiländerung (Ernährung und Bewegung) mit dem Ziel einer Gewichtsreduktion von 7 % zu untersuchen [40]. In der Interventionsgruppe war der Gewichtsverlust nach 4 Jahren durchschnittlich 4,7 % vs. 0,8 % in der Kontrollgruppe, aber nur wenige (7 % vs. 2 %) hatten einen HbA_1c_-Wert < 6,5 % [41]. Dies deutet darauf hin, dass eine größere Gewichtsabnahme erforderlich ist, um einen signifikanten Einfluss auf den Krankheitsverlauf von Typ-2-Diabetes zu erzielen. Interessanterweise war nach 10 Jahren der primäre Endpunkt der kardiovaskulären Ereignisse zwischen den Gruppen nicht signifikant unterschiedlich. Allerdings zeigte eine Post-hoc-Analyse, dass die 21 % der Teilnehmer:innen, die im ersten Jahr mindestens 10 % ihres Körpergewichts verloren hatten, über einen Zeitraum von 10 Jahren ein um 21 % geringeres Risiko für kardiovaskuläre Ereignisse hatten als Personen mit stabilem Gewicht oder Gewichtszunahme [42].

In der DiRECT-Studie erfolgte eine intensive Ernährungsintervention bei 306 Menschen (BMI 27–45 kg/m^2^, mittlerer HbA_1c_ 7,7 %) mit Typ-2-Diabetes (Erkrankungsdauer < 6 Jahre) [5] im allgemeinmedizinischen Bereich. In einer Analyse hatten 70 % der Personen (*n* = 20), die mindestens 15 kg abgenommen hatten, eine Remission des Diabetes nach 2 Jahren [6]. Sechzig Prozent der Personen (*n* = 25), die zwischen 10 und 15 kg, 29 % der Personen (*n* = 79), die zwischen 5 und 10 kg und 5 % der Teilnehmer:innen (*n* = 154), die < 5 kg abgenommen hatten, wiesen nach 2 Jahren eine Remission ihres Diabetes auf (insgesamt 36 % der Teilnehmer:innen in der Interventionsgruppe). Personen in der intensiven Interventionsgruppe, bei denen keine Remission von Typ-2-Diabetes eintrat, zeigten dennoch eine größere HbA_1c_ und Gewichtsreduktion und benötigten weniger blutzuckersenkende Medikamente als die Kontrollgruppe. Den Teilnehmer:innen der Interventionsgruppe wurde angeboten, weitere 3 Jahre zu verlängern, hier wurden sie nochmals in eine DiRECT-Extension- und Non-Extension-Studie randomisiert (2:1) [43]. Die Extension-Studie beinhaltete weitere diätologische Betreuung der Teilnehmer:innen. Nach 5 Jahren lag der Gewichtsverlust im Mittel bei 6,1 kg, allerdings waren nur mehr 13 % (*n* = 11) der Teilnehmer:innen nach 5 Jahren in einer Diabetesremission [43]. Diese Daten zeigen einerseits, dass trotz anhaltenden Gewichtsverlusts die Remissionsrate über die Jahre geringer wird, andererseits, dass trotz zu Beginn intensiver Lebensstilmodifikation und danach folgender langfristiger diätologischer Begleitung nur ein moderater mittlerer Gewichtsverlust von 6,1 kg über 5 Jahre erreichbar ist.

Es scheint wichtig festzuhalten, dass jede Form der Lebensstilveränderung nur dann langfristig eine Gewichtsnormalisierung ermöglicht, wenn die Veränderung dauerhaft umgesetzt und als eine neue Normalität verstanden wird.

## Medikamentöse Adipositastherapie bei Menschen mit Typ-2-Diabetes

In Bezug auf gewichtsreduzierende und auch blutzuckersenkende Wirkung hat mit den Inkretinmimetika ein neues Zeitalter begonnen, welches auch das pathophysiologische Verständnis von Adipositas und Diabetes deutlich verändert und erweitert hat. Kürzlich veröffentlichte die EASO ein Framework zur Behandlung von Menschen mit Adipositas mit verschiedenen Begleiterkrankungen unter anderem auch Typ-2-Diabetes [44]. Abhängig von der Potenz der Inkretinmimetika bezüglich Gewichtsreduktion und Blutzuckersenkung ergibt sich folgende Reihung: Tirzepatid, Semaglutid und dann Liraglutid. Das Konsensuspapier der Österreichischen Adipositasgesellschaft unterscheidet nicht nach Begleiterkrankungen, dementsprechend werden die Medikamente auch nicht gereiht [45].

### Tirzepatid

Der duale GIP/GLP-1-Rezeptoragonist Tirzepatid wurde im SURPASS-Studienprogramm hinsichtlich einer Verbesserung des Typ-2-Diabetes und im SURMOUNT-Studienprogramm der Adipositas untersucht. Er ist in den Dosierungen 5 mg, 10 mg, 15 mg sowohl zur Behandlung von Typ 2 als auch zur Behandlung von Adipositas zugelassen. In der SURPASS 2-Studie wurden 5 mg, 10 mg und 15 mg Tirzepatid pro Woche mit 1 mg Semaglutid wöchentlich verglichen. Letztlich konnte für 10 mg bzw. 15 mg Tirzepatid eine klare Überlegenheit im Vergleich zu Semaglutid sowohl hinsichtlich HbA_1c_ (*p* = 0,002) als auch Gewichtsreduktion (*p* < 0,001) gezeigt werden [46]. Interessant ist die SURMOUNT 2-Studie, welche als primären Endpunkt den Gewichtsverlust bei Menschen mit Typ-2-Diabetes hatte [47]. In der Studie fand im Gegensatz zum SURPASS-Studienprogramm eine spezifische diätologische Betreuung statt. Der Gewichtsverlust in der Surmount 2-Studie war in der 15-mg-Tirzepatid-Gruppe mit 15,6 % deutlich größer als z. B. in der 15-mg-Tirzepatid-Gruppe der SURPASS 3-Studie (11,3 %), ein deutlicher Hinweis, wie relevant zusätzliche Lebensstilmaßnahmen sind [48]. In der Surmount 5-Studie wurde Tirzepatid (maximal tolerierbare Dosis 10 mg oder 15 mg) mit Semaglutid (maximal tolerierbare Dosis 1,7 mg oder 2,4 mg) zur Behandlung von Adipositas verglichen. Tirzepatid erzielte 20,2 % vs. Semaglutid 13,7 % Gewichtsverlust nach 72 Wochen [49].

In der Verlängerung der Surmount 1-Studie über 3 Jahre konnte eine Therapie mit Tirzepatid bei Menschen mit Adipositas und Prädiabetes die Inzidenz von Typ-2-Diabetes um 93 % reduzieren [4].

### Semaglutid

Semaglutid ist als Semaglutid 2,4 mg für die Behandlung von Adipositas zugelassen und wird 1‑mal wöchentlich injiziert. In der STEP 2-Studie (1210 Menschen mit Diabetes mellitus Typ 2) wurde Semaglutid 1 mg vs. 2,4 mg Semaglutid zusätzlich zur lebensstilmodifizierenden Therapie verglichen. Nach 68 Wochen erreichten die Patient:innen in der 2,4-mg-Gruppe einen mittleren Gewichtsverlust von 9,6 % verglichen mit 3,4 % in der Gruppe mit 1 mg. Zwischen den beiden Gruppen zeigte sich kein Unterschied hinsichtlich der HbA_1c_ Senkung [50].

In der STEP 10-Studie wurde bei Menschen mit Adipositas und Prädiabetes (BMI 40,1 kg/m^2^; HbA_1c_ 5,9 %) Semaglutid 2,4 mg vs. Placebo hinsichtlich Normoglykämie und Gewichtsverlust untersucht [51]. Nach 52 Wochen zeigte sich ein Gewichtsverlust in der Semaglutid-Gruppe von 13,9 % vs. 2,7 % mit Placebo sowie eine Rückkehr zur normalen Glukosetoleranz von 81 % vs. 14 % in der Placebogruppe. Die STEP Teens-Studie zeigte bei Kindern zwischen 12 und 18 Jahren mit Adipositas, dass 2,4 mg Semaglutid den BMI um 16,1 % erfolgreich senken konnten [52]. Erwähnt sei an dieser Stelle, dass Semaglutid 2,4 mg bei Menschen ohne Diabetes mellitus Typ 2 (in dieser Studie waren allerdings 66 % der Teilnehmer:innen von Prädiabetes betroffen) eine Reduktion des 3 Punkt-MACE in einer kardiovaskulären Endpunktstudie verglichen zu Placebo um 20 % erzielen konnte [53]. Dies ist die erste positive Endpunktstudie in einer Gruppe von kardiovaskulär erkrankten Menschen mit Übergewicht/Adipositas, aber ohne Typ-2-Diabetes, die eine Reduktion von nichttödlichem Myokardinfarkt, nichttödlichem Schlaganfall und kardiovaskulärem Tod mit einer Therapie mit einem GLP-1-Rezeptoragonisten zeigt.

### Liraglutid

Im Rahmen des SCALE-Studienprogrammes wurde Liraglutid 3,0 mg zur Behandlung von Menschen mit Adipositas in Kombination mit einer kalorienreduzierten Diät und Bewegung untersucht. In der SCALE Diabetes-Studie (623 Patient:innen mit Diabetes mellitus Typ 2) erreichten die Teilnehmer:innen einen mittleren Gewichtsverlust von 6 % nach einem Jahr Behandlungsdauer, bei 25 % war der Gewichtsverlust sogar 10 % oder größer [2]. Bei Menschen mit Prädiabetes erreichte Liraglutid eine 79 %ige Risikoreduktion für eine Manifestation mit Typ-2-Diabetes über einen Zeitraum von 3 Jahren [54].

Liraglutid 3,0 mg ist bei Jugendlichen ab 12 Jahren mit Adipositas (BMI ≥ 30 kg/m^2^ für Erwachsene gemäß internationalen alters- und geschlechtsbezogenen Grenzwerten), die mehr als 60 kg wiegen, sowie bei Kindern von 6 bis unter 12 Jahren mit Adipositas (BMI ≥ 95. Perzentile), die mindestens 45 kg wiegen, zur Behandlung der Adipositas zugelassen [55].

Nebenwirkungen der GLP-1- sowie GIP/GLP-1-Rezeptoragonisten-Therapie waren v. a. gastrointestinaler Natur, wie genauer im Kapitel antihyperglykämische Therapie des Typ-2-Diabetes beschrieben. In Bezug auf die etwas höheren Raten an Cholezystolithiasis ist hervorzuheben, dass jede Art von Gewichtsverlust zu einer Zunahme von Cholezystolithiasis führen kann und es sich daher vermutlich nicht um eine substanzspezifische Nebenwirkung der Inkretinmimetika handelt [56].

Der Triglyzerid-Lipase-Hemmer Orlistat ist als eine rezeptpflichtige Formulierung und eine OTC-Formulierung am Markt. Orlistat soll gleichzeitig mit einer fettarmen Diät eingesetzt werden (die selbst eine Lebensstiltherapie mit Gewichtsreduktion unterstützt) und reduziert die Fettresorption um weitere 30 % [57]. Potenzielle Nebenwirkungen, insbesondere wenn eine fettreiche Ernährung die Norm ist, sind in erster Linie gastrointestinaler Natur (Fettstühle, Diarrhöen). Außerdem ist als weitere orale Therapieform die Fix-Kombination von Naltrexon und Bupropion in Europa von der EMA zugelassen [58]. Beide Pharmaka sind seit 2018 auch in Österreich zur Verwendung in der Adipositastherapie erhältlich. Beide Präparate zeigten auch in Studien bei Menschen mit Typ-2-Diabetes einen moderaten Gewichtsverlust sowie eine HbA_1c_-Verbesserung [59, 60].

## Metabolische/bariatrische Chirurgie

Bariatrische Operationen werden, wenn das Ziel die Verbesserung einer metabolischen Erkrankung ist, als metabolische Operation bezeichnet [61]. Generell kann durch eine metabolische Operation eine 15- bis 40 %-Reduktion des Ausgangsgewichtes erreicht werden [62], wodurch sich Adipositas-assoziierte Komorbiditäten wie Diabetes verbessern und die Gesamtmortalität reduziert wird [63–65]. Derzeit ist die Indikation für bariatrische Operationen bei Menschen mit Adipositas ohne Diabetes bei einem BMI > 35 kg/m^2^ und bei Menschen mit Typ-2-Diabetes bei einem BMI > 30 kg/m^2^ gegeben [66]. Auch wenn dadurch die bariatrische Chirurgie einer immer größeren Population zugänglich gemacht wird, sollte die Wahl der Patient:innen äußerst gewissenhaft erfolgen, damit die erforderlichen Basis- und Kontrolluntersuchungen vor und nach bariatrischen Operationen erfolgen können. Für die sehr umfangreiche multidisziplinäre medizinische Nachbetreuung (insbesondere Mikro- und Makronährstoffmängel sowie das Dumping-Syndrom) dieser Patient:innen verweisen wir auf das Konsensuspapier der Österreichischen Adipositasgesellschaft [67]. Die bariatrische Chirurgie gilt auch als mögliche Therapieoption für Typ-2-Diabetes, da sie sowohl zu einer signifikanten Verbesserung der Hyperglykämie beitragen als auch zu einer kompletten Diabetesremission führen kann [68, 69]. Die Diabetesremissionsrate beträgt 45–95 %, je nachdem, welche Operation durchgeführt wurde, wie lange der Diabetes besteht und welcher Therapiebedarf/Insulinbedarf initial benötigt wird – wobei ein größerer Gewichtsverlust zu einer höheren Diabetesremission beiträgt [7, 70, 71]. In einer randomisierten Studie, der STAMPEDE-Studie, konnte gezeigt werden, dass bariatrische Operationen bei Menschen mit Typ-2-Diabetes einer rein medikamentösen Therapie hinsichtlich Gewichtsreduktion, glykämischer Kontrolle und Gebrauch von antidiabetischer, antihypertensiver sowie lipidsenkender Medikation überlegen sind [7]. Die positiven Effekte blieben auch noch 5 Jahre nach der Operation erhalten [7]. Allerdings kann es einige Jahre nach bariatrischer/metabolischer Operation zu einem Wiederauftreten des Diabetes mellitus Typ 2 bei gleichzeitiger erneuter Gewichtszunahme kommen. Hier ist ein engmaschiges Monitoring wichtig, verschiedene Möglichkeiten sollten in Erwägung gezogen werden, um den erneuten Gewichtsverlust zu vermeiden bzw. gering zu halten [72]. Auch hier gibt es gute Ergebnisse mit Inkretinmimetika, sodass eine solche Therapie angedacht werden sollte [73].

## Interessenkonflikt

J.M. Brix hat von folgenden Unternehmen, die z. T. auch fördernde Mitglieder der ÖDG sind, Forschungsunterstützungen und/oder Honorare erhalten: Abbott, AstraZeneca, Bayer, Boehringer Ingelheim, Dexcom, Eli Lilly, Medtronic, Novo Nordisk. M. Clodi hat für Vorträge, Studien und Beratung Honorare von Novo Nordisk, Elli Lilly, Boehringer Ingelheim, AstraZeneca, Novartis, Daiichi Sankyo, Amgen, Wörwag, Sanofi, Amarin, Roche, Bayer erhalten. J. Harreiter hat von folgenden Unternehmen Forschungsunterstützungen und/oder Honorare erhalten: Amgen, AstraZeneca, Bayer, Boehringer Ingelheim, Novartis, Novo Nordisk, Ultragenyx. F. Hoppichler hat von folgenden Unternehmen, die z. T. auch fördernde Mitglieder der ÖDG sind, Honorare für Beratungs- oder Vortragstätigkeit erhalten: Boehringer Ingelheim, Eli Lilly, Novo Nordisk, Sanofi. F.W. Kiefer hat von folgenden Unternehmen, die z. T. auch fördernde Mitglieder der ÖDG sind, Honorare für Beratungs- oder Vortragstätigkeit erhalten: Eli Lilly und Novo Nordisk. B.K. Itariu wurde für Beratungs- und Vortragstätigkeit von Amgen, Boehringer Ingelheim, Novo Nordisk und Eli Lilly honoriert. V. Parzer hat von folgenden Unternehmen, die z. T. auch fördernde Mitglieder der ÖDG sind, Honorare für Beratungs- oder Vortragstätigkeit erhalten: Eli Lilly und Novo Nordisk. M. Resl hat von folgenden Unternehmen, die z. T. auch fördernde Mitglieder der ÖDG sind, Forschungsunterstützungen und/oder Honorare erhalten: Boehringer Ingelheim, Eli Lilly, Dexcom, Abbott, Roche, Bayer. K. Schindler hat keine Interessenkonflikte anzugeben. H. Toplak gibt Unterstützung klinischer Studien und medizinischer Beirat sowie Referentenhonorare durch Novo Nordisk an, Honorare für medizinische Beratung durch Kwizda. T.C. Wascher Honorare für Fortbildung und Beratung von Novo Nordisk und Eli Lilly. B. Ludvik hat von folgenden Unternehmen, die z. T. auch fördernde Mitglieder der ÖDG sind, Forschungsunterstützungen und/oder Honorare erhalten: Amarin, Amgen, AstraZeneca, Bayer, Boehringer Ingelheim, Daiichi Sankyo, Eli Lilly, Novo Nordisk.
